# Histopathological Findings Predict Renal Recovery in Severe ANCA-Associated Vasculitis Requiring Intensive Care Treatment

**DOI:** 10.3389/fmed.2020.622028

**Published:** 2021-02-09

**Authors:** Samy Hakroush, Desiree Tampe, Peter Korsten, Philipp Ströbel, Michael Zeisberg, Björn Tampe

**Affiliations:** ^1^Institute of Pathology, University Medical Center Göttingen, Göttingen, Germany; ^2^Department of Nephrology and Rheumatology, University Medical Center Göttingen, Göttingen, Germany; ^3^German Center for Cardiovascular Research (DZHK), Göttingen, Germany

**Keywords:** autoimmune diseases, systemic vasculitis, inflammation, ANCA-associated vasculitis, acute kidney injury, renal replacement therapy, intensive care treatment

## Abstract

Renal involvement is a common and severe complication of AAV as it can cause ESRD. Histopathological subgrouping and ARRS are helpful to predict long-term ESRD in patients with AAV. Because a subgroup of critically ill patients with severe AAV present with deterioration of kidney function requiring RRT at admission, we here aimed to evaluate histopathological findings and predictive value of Berden's histopathological subgrouping and ARRS for severity of AKI and requirement of RRT during the short-term clinical course in critically ill patients requiring intensive care treatment and predictors for short-term renal recovery in patients requiring RRT. A subgroup of 15/46 (32. 6%) AAV patients with biopsy-proven AAV required RRT during the short-term course of disease, associated with requirement of critical care treatment. While histopathological subgrouping and ARRS were associated with requirement of acute RRT, presence of global glomerular scarring was the strongest predictor of failure to recover from RRT after initiation of remission induction therapy. This new aspect requires further investigation in a prospective controlled setting for therapeutic decision making especially in this subgroup.

## Introduction

Anti-neutrophil cytoplasmic antibody (ANCA)-associated vasculitis (AAV) is a systemic vasculitis, which most frequently presents as microscopic polyangiitis (MPA) or granulomatosis with polyangiitis (GPA) (1). Renal involvement is a common and severe complication of AAV as it can cause end-stage renal disease (ESRD) or death (2, 3). Histopathological subgrouping into four classes (focal, crescentic, mixed, and sclerotic) as defined by Berden et al. was proposed to predict long-term renal survival rates poorest in the sclerotic class (sclerotic glomeruli above 50%) (4). Unlike Berden's classification, Brix et al. suggested the ANCA renal risk score (ARRS) by incorporation of the baseline glomerular filtration rate (GFR) to the histopathological findings (percentage of normal glomeruli, tubular atrophy/interstitial fibrosis) to predict ESRD in patients with AAV (5). Histopathological subgrouping and ARRS were both established for predicting long-term ESRD over years, but a subgroup of severe AAV presents with acute kidney injury (AKI) required renal replacement therapy (RRT) during the initial course of the disease (4, 5). Since severity of AKI, requirement of RRT, and short-term renal recovery in critically ill patients are associated with disease severity and clinical course of disease, predictors for RRT requirement and renal recovery after initiation of remission induction therapy are of relevance (6). Therefore, we here aimed to evaluate the histopathological findings and predictive value of Berden's histopathological subgrouping and ARRS for severity of AKI and requirement of RRT during the short-term clinical course in critically ill patients requiring intensive care treatment. In addition, we sought to identify predictors for short-term renal recovery in patients requiring RRT.

## Methods

### Study Population

A total number of 46 patients with biopsy-proven AAV at the University Medical Center Göttingen were retrospectively included between 2015 till 2020. While no formal approval was required for the use of routine clinical data, a favorable ethical opinion was granted by the local Ethics committee (no. 4/8/19). A detailed Strengthening the Reporting of Observational Studies in Epidemiology (STROBE) flowchart of patient disposition is shown in Supplementary Figure 1A.

### Definitions

At admission, the Birmingham Vasculitis Activity Score (BVAS) version 3 was calculated as described previously (7). The simplified acute physiology score (SAPS) II and estimated mortality rates were calculated according to the published guidelines (8). Requirement of intensive care treatment was defined at admission and calculated by the time between admission to the intensive care unit (ICU) or intermediate care unit (IMC) and relocation to the non-ICU/non-IMC medical ward; all patients required critical care treatment > 24 h. RRT was performed intermittently in all cases. Indications of RRT included serum creatinine ≥ 500 μmol/L, severe electrolyte and acid–base abnormalities, volume overload, and encephalopathy. RRT was terminated when the glomerular filtration rate (GFR) according to CKD-EPI surpassed 15 mL/min/1.73 m^2^ and there was no hyperkalemia, heart failure, edema, and encephalopathy. Short-term course of disease was defined within 30 days after admission; short-term renal recovery was defined as successful recovery from RRT within 30 days after RRT initiation.

### Renal Histopathology

Renal pathologists (SH and PS) evaluated the biopsies. Within a kidney biopsy specimen, each glomerulus had to be scored separately for the presence of necrosis, crescents, and global sclerosis (Supplementary Figure 2A). Consequently, the percentage of glomeruli with any of these features was calculated as a fraction of the total number of glomeruli in the biopsy. Apart from these categories, degree of interstitial fibrosis/tubular atrophy (IF/TA) was quantified. Based on these scorings, histopathological subgrouping according to Berden et al. (focal, crescentic, mixed, or sclerotic class) and ARRS according to Brix et al. (low, medium, or high risk) were performed (4, 5).

### Remission Induction Therapy

Glucocorticoids (GCs) were administered either as intravenous pulse therapy or orally with a tapering schedule. Plasma exchange (PEX) was administered during the induction period at the discretion of treating physicians. Rituximab (RTX) was administered as four intravenous doses at 375 mg/m^2^ every week; RTX was not administered within 48 h before PEX treatment. Cyclophosphamide (CYC) was administered as three intravenous doses up to 15 mg/kg every 2 weeks and every 3 weeks thereafter, adjusted for age and renal function. Combination therapy was administered as four intravenous doses at 375 mg/m^2^ RTX every week and two intravenous doses at 15 mg/kg CYC every 2 weeks. On the discretion of treating physicians, choice of remission induction therapy was dependent on previous regimens and individual patients, more likely to choose RTX in younger patients with toxicity being the main reason for this choice (9). Prophylaxis to prevent pneumocystis (carinii) jiroveci infection was administered according to local practice.

### Statistical Methods

Variables were tested for normal distribution using the Shapiro–Wilk test. Non-normally distributed continuous variables are expressed as median and interquartile range (IQR); categorical variables are presented as frequency and percentage. Statistical comparisons were not formally powered or prespecified. For group comparisons, the Mann–Whitney *U*-test was used to determine differences in medians. Non-parametric between-group comparisons were performed with Pearson's chi-square test. To establish a cutoff for each parameter, the ability of prognostic factors to discriminate groups was evaluated by receiver operator curves (ROC) and the area under the curve (AUC), as well as sensitivity and specificity for prediction. An AUC of 1.0 indicates perfect concordance, an AUC of 0.50 would indicate that the ability of prognostic factors to discriminate groups is no better than chance. Sensitivity and specificity were based on selection of the cutoff point on the ROC that maximized Youden's index (sensitivity+specificity-1), comparison of survival curves was performed with log rank (Mantel–Cox) testing (10). Data analyses were performed with GraphPad Prism (version 8.4.0 for MacOS, GraphPad Software, San Diego, California, USA).

## Results

**Figure 1:**
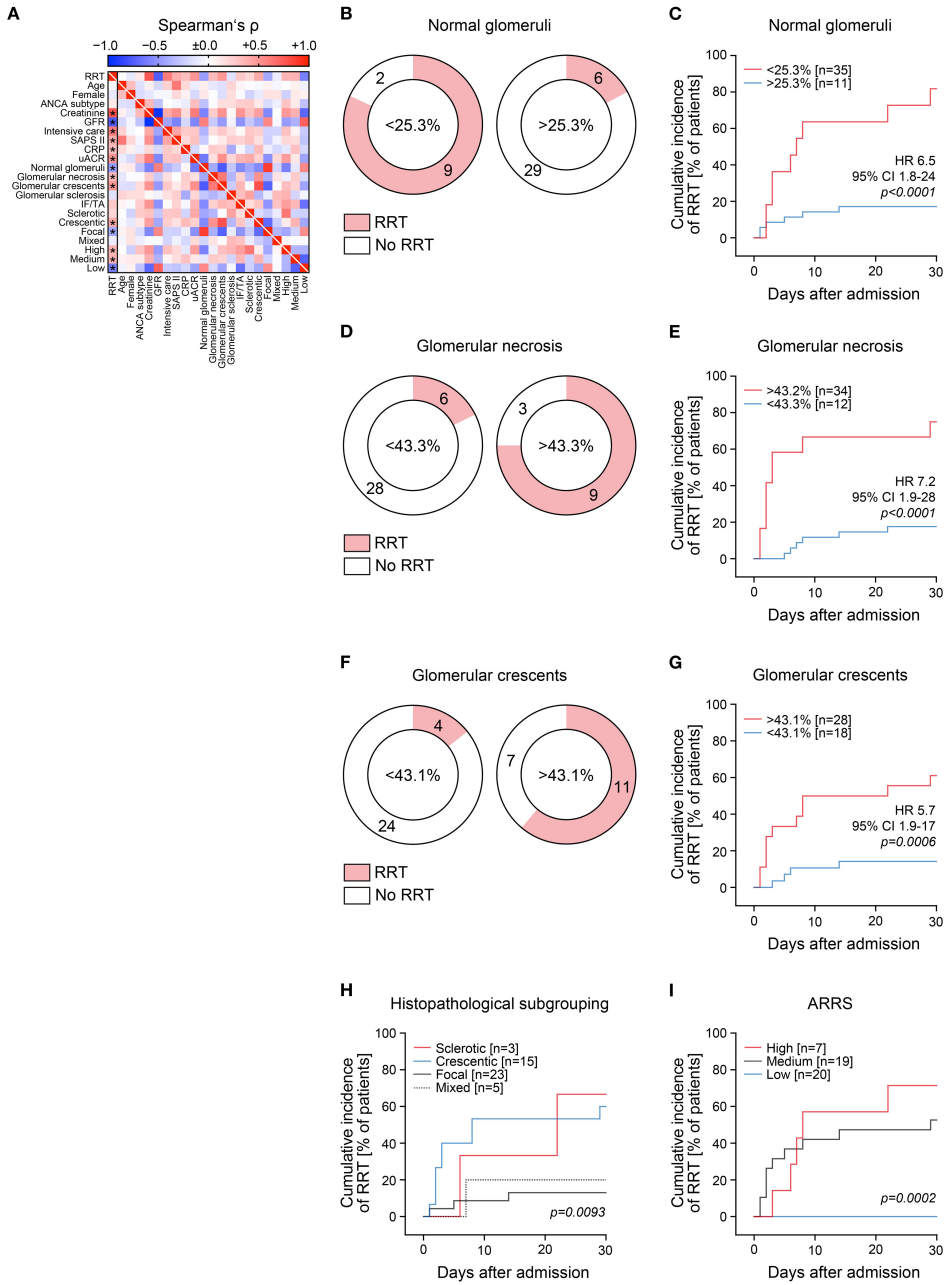
Histopathological findings associate with severity of acute kidney injury in severe AAV. (**A**) Association between requirement of RRT within 30 days after admission; clinical/laboratory and histopathological findings is shown by a heat map reflecting mean values of Spearman's ρ, asterisks indicate *p* < *0.05*. (**B–G**) Cutoff points on the ROC that maximized Youden's index were used for cumulative incidence of RRT within 30 days after admission for each parameter. (**H,I**) Histopathological subgrouping and ARRS for successful recovery from RRT within 30 days are shown. Analysis was performed using log rank (Mantel–Cox) testing. ANCA, anti-neutrophil cytoplasmic antibodies; ARRS, ANCA renal risk score; CI, confidence interval; CRP, C-reactive protein; GFR, glomerular filtration rate (CKD-EPI); HR, hazard ratio; IF/TA, interstitial fibrosis/tubular atrophy; RRT, renal replacement therapy; SAPS II, simplified acute physiology score II; uACR, urinary albumin/creatinine ratio.

During the short-term course of the disease, 15/46 (32.6%) patients presented with severe AKI requiring RRT within 30 days after admission (Supplementary Figure 1A). Requirement for RRT was associated with disease severity reflected by significantly higher SAPS II at admission, level of intensive care treatment longer than 24 h, prolonged requirement of intensive care treatment, higher levels of C-reactive protein, and urinary albumin/creatinine ratio (uACR, Figure 1A and Table 1). We next scored each glomerulus for the presence of necrosis, crescents, and global sclerosis (Supplementary Figure 2A). Renal histology revealed that requirement of RRT during the short-term course of disease was associated with active glomerular lesions reflected by lower number of normal glomeruli, increased glomerular necrosis, and crescents (Figure 1A and Table 1). ROC analysis confirmed an association between RRT requirement and amounts of normal glomeruli (AUC 0.7860, *p* = *0.0018*), glomerular necrosis (AUC 0.6989, *p* = *0.0302*), and crescents (AUC 0.7946, *p* = *0.0013*, Supplementary Figures 3A–C). Renal survival analysis for cumulative incidence of RRT by using the cutoff point that maximized the combined sensitivity and specificity (Youden's index) of each parameter confirmed the poorest short-term renal outcome in patients with lower number of normal glomeruli, increased glomerular necrosis, and crescents (Figures 1B–G). Histopathological subgrouping into sclerotic and crescentic classes along with ARRS high-risk classification were associated with RRT requirement during short-term disease course (Table 1 and Figures 1H,I), both considered to also show poorest long-term renal survival rates (4, 5).

**Table 1:** Characteristics of patients: requirement of RRT vs. no RRT within 30 days after admission.

	**RRT (*n* = 15)**	**No RRT (*n* = 31)**	***P*-value**
Median age (IQR)—years	66 (50–76)	63 (53–74)	*0.9676*
Female sex—no. (%)	5 (33.3)	14 (45.2)	*0.4450*
ANCA subtype MPO/PR3—no. (%)	9/6 (60/40)	15/16 (48.4/51.6)	*0.4598*
History of vasculitis—no. (%)	1 (6.7)	5 (16.1)	*0.3717*
**Renal injury**			
Median serum creatinine (IQR)—μmol/LSerum creatinine ≥ 500 μmol/liter—no. (%)Median GFR (IQR)—mL/min/1.73 m^2^	567 (438–652) 10 (66.7) 8.4 (5.8–9.6)	155 (99–280)0 (0)32.2 (15.3–60.9)	*** < 0.0001*** *** < 0.0001*** *** < 0.0001***
**Extrarenal manifestations**			
Pulmonary hemorrhage—no. (%)Skin involvement—no. (%)	2 (13.3) 2 (13.3)	4 (12.9) 6 (19.4)	*0.9676* *0.6135*
**Disease activity**			
Median BVAS (IQR)—pointsMedian SAPS II at admission (IQR)—pointsIntensive care treatment—no. (%)Median intensive care treatment (IQR)—days	18 (18–20) 31 (24–35) 14 (93.3) 5 (3–10)	17 (14–21) 23 (19–30) 9 (29) 0 (0–3)	*0.2031* ***0.0085*** *** < 0.0001*** *** < 0.0001***
Median CRP (IQR)—mg/LMedian uACR (IQR)—mg/g	73.6 (49.3–174) 839 (678–2,246)	37 (10.8–89) 243 (100–481)	***0.0382*** ***0.0006***
**Renal histology**			
Median total glomeruli (IQR)—no.Median normal glomeruli (IQR)—no.Median normal glomeruli (IQR)—%Median glomerular necrosis (IQR)—no.Median glomerular necrosis (IQR)—%Median glomerular crescents (IQR)—no.Median glomerular crescents (IQR)—%Median glomerular sclerosis (IQR)—no.Median glomerular sclerosis (IQR)—%Median IF/TA (IQR)—%	15 (10–18) 3 (1–9) 20 (11.1–45.5) 7 (0–15) 46.7 (0–80) 8 (5–15) 54.6 (33.3–80) 0 (0–5) 0 (0–33.3) 30 (10–50)	17 (11–28)10 (5–14)58.8 (37–80)2 (1–5)12.5 (3.6–28)2 (1–11)27.3 (0–42.4)2 (0–3)9.4 (0–23.1)15 (7.5–30)	*0.5814* ***0.0044*** ***0.0013*** *0.0726* ***0.0284*** ***0.0389*** ***0.0009*** *0.4074* *0.3438* *0.0839*
**Follow-up**			
Median follow-up (IQR)—daysDeath—no. (%)RRT—no. (%)	386 (165–715) 1 (6.7) 4 (26.7)	300 (81–609)2 (6.5)1 (3.2)	*0.5779* *0.9779* ***0.0166***

*For group comparisons, the Mann–Whitney U-test was used to determine differences in medians. Non-parametric between-group comparisons were performed with Pearson's chi-square test. Bold indicates statistically significant values at group level. ANCA, anti-neutrophil cytoplasmic antibodies; BVAS, Birmingham Vasculitis Activity Score; CRP, C-reactive protein; GFR, glomerular filtration rate (CKD-EPI); IF/TA, interstitial fibrosis/tubular atrophy; IQR, interquartile range; No., number; MPO, myeloperoxidase; PR3, proteinase 3; RRT, renal replacement therapy; RTX, rituximab; SAPS II, simplified acute physiology score II; uACR, urinary albumin/creatinine ratio*.

**Figure 2:**
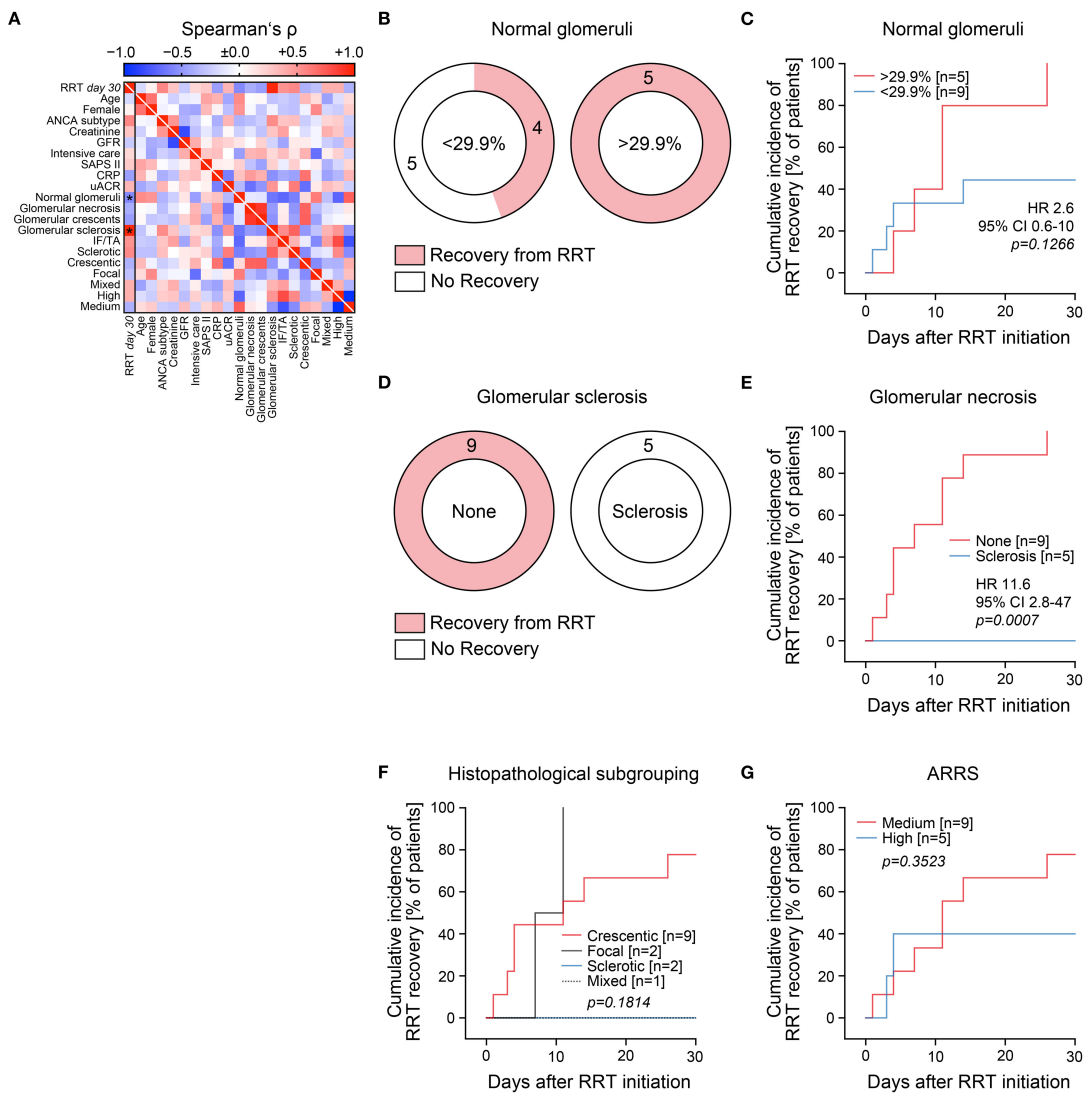
Global glomerular sclerosis associates with failure to recover from RRT in critically ill patients. **(A)** Association between successful recovery from RRT within 30 days after RRT initiation; clinical/laboratory and histopathological findings are shown by heat map reflecting mean values of Spearman's ρ, asterisks indicate *p* < *0.05*. (**B–E**) Cutoff points on the ROC that maximized Youden's index were used for cumulative incidence of successful recovery from RRT within 30 days after RRT initiation for each parameter. (**F,G**) Histopathological subgrouping and ARRS for successful recovery from RRT within 30 days after RRT initiation are shown. Analysis was performed using log rank (Mantel–Cox) testing. ANCA, anti-neutrophil cytoplasmic antibodies; ARRS, ANCA renal risk score; CI, confidence interval; CRP, C-reactive protein; GFR, glomerular filtration rate (CKD-EPI); HR, hazard ratio; IF/TA, interstitial fibrosis/tubular atrophy; RRT, renal replacement therapy; SAPS II, simplified acute physiology score II; uACR, urinary albumin/creatinine ratio.

After initiation of remission induction therapy, we next analyzed predictors for short-term renal recovery reflected by successful recovery from RRT within 30 days after RRT initiation. After one dropout due to death at day 21 after admission not included in the further analysis, 9/14 (64.3%) critically ill patients that required RRT during the course of disease recovered within 30 days after initiation of RRT (Supplementary Figure 1A). Among all parameters analyzed, short-term recovery from RRT after initiation of remission induction therapy was associated with higher numbers of unaffected glomeruli, whereas presence of sclerotic glomeruli was the strongest negative predictor (Figure 2A and Table 2). ROC analysis confirmed superiority of global glomerular sclerosis to predict failure for short-term renal recovery (AUC 1.000, *p* = *0.0027*, Supplementary Figures 4A,B), further supported by renal survival analysis for RRT requirement (Figures 2B–E). In contrast, histopathological subgrouping and ARRS both failed to identify patients that successfully recovered from RRT during short-term disease course (Figures 2F,G and Table 2). While only limited follow-up data was available, renal outcome within 30 days significantly correlated with long-term renal survival rates (Table 2).

**Table 2:** Characteristics of patients requiring RRT: short-term recovery vs. no recovery from RRT within 30 days.

	**Recovery (*n* = 9)**	**No recovery (*n* = 5)**	***P*-value**
Median age (IQR)—years	66 (52.5–73.5)	58 (43–73)	*0.5400*
Female sex—no. (%)	3 (33.3)	2 (40)	*0.8030*
ANCA subtype MPO/PR3—no. (%)	4/5 (44.4/55.6)	5/0 (100/0)	***0.0376***
History of vasculitis—no. (%)	0 (0)	1 (20)	*0.1638*
**Renal injury**			
Median serum creatinine (IQR)—μmol/LSerum creatinine ≥ 500 μmol/liter—no. (%)Median GFR (IQR)—mL/min/1.73 m^2^	567 (423–640) 6 (66.7) 8.4 (6.3–9.7)	643 (473–900)4 (80)8 (4.6–9.7)	*0.3636* *0.5967* *0.3816*
**Extrarenal manifestations**			
Pulmonary hemorrhage—no. (%)Skin involvement—no. (%)	0 (0) 2 (22.2)	2 (40)0 (0)	***0.0404*** *0.2549*
**Disease activity**			
Median BVAS (IQR)—pointsMedian SAPS II at admission (IQR)—pointsIntensive care treatment—no. (%)Median intensive care treatment (IQR)—days	18 (18–19) 30 (24–33.5) 8 (88.9) 4 (3.5–7)	18 (16.5–23)35 (22–50)5 (100)8 (2–23)	*0.7657* *0.3771* *0.4392* *0.5400*
Median CRP (IQR)—mg/LMedian uACR (IQR)—mg/g	77.3 (68.1–148) 839 (520–1,143)	49.3 (24–120)2,849 (547–3,736)	*0.1119* *0.2398*
**Renal histology**			
Median total glomeruli (IQR)—no.Median normal glomeruli (IQR)—no.Median normal glomeruli (IQR)—%Median glomerular necrosis (IQR)—no.Median glomerular necrosis (IQR)—%Median glomerular crescents (IQR)—no.Median glomerular crescents (IQR)—%Median glomerular sclerosis (IQR)—no.Median glomerular sclerosis (IQR)—%Median TA/IF (IQR)—%	16 (9.5–22.5) 4 (2–8.5) 35.3 (11.4–50.9) 8 (3.5–18.5) 64.7 (21.9–88.6) 8 (6.5–18.5) 64.7 (49.2–88.6) 0 (0–0) 0 (0–0) 20 (10–37.5)	15 (9.5–32)1 (0–7.5)6.7 (0–22.2)3 (0–21)20 (0–59.1)5 (3–21.5)45.5 (22.9–62.4)7 (3.5–9.5)46.7 (18.7–100)30 (25–75)	*0.9171* *0.2048* ***0.0490*** *0.3751* *0.1748* *0.3801* *0.1064* ***0.0005*** ***0.0005*** *0.0784*
**Remission induction therapy**			
Intravenous steroid pulse—no. (%)Oral GCs—no. (%)	6 (66.7) 9 (100)	5 (100)5 (100)	
PEX—no. (%)Median sessions of PEX (IQR)—no.	8 (88.9) 5 (4–6)	3 (60)5 (5–8)	
RTX—no. (%)CYC—no. (%)RTX/CYC—no. (%)	1 (11.1) 5 (55.6) 3 (33.3)	2 (40)3 (60)0 (0)	
**Long-term survival**			
Median follow-up (IQR)—daysDeath—no. (%)RRT—no. (%)	505 (280–880) 0 (0) 0 (0)	326 (37–920)0 (0)3 (60)	*0.2977* ***0.0088***

*For group comparisons, the Mann–Whitney U-test was used to determine differences in medians. Non-parametric between-group comparisons were performed with Pearson's chi-square test. Bold indicates statistically significant values at group level. ANCA, anti-neutrophil cytoplasmic antibodies; BVAS, Birmingham Vasculitis Activity Score; CRP, C-reactive protein; CYC, cyclophosphamide; GCs, glucocorticoids; GFR, glomerular filtration rate (CKD-EPI); IF/TA, interstitial fibrosis/tubular atrophy; IQR, interquartile range; No., number; MPO, myeloperoxidase; PEX, plasma exchange; PR3, proteinase 3; RRT, renal replacement therapy; RTX, rituximab; SAPS II, simplified acute physiology score II; uACR, urinary albumin/creatinine ratio*.

## Discussion

Histopathological subgrouping and ARRS are helpful for risk stratification to predict long-term renal survival rates (4, 5). While these observations have been validated in independent cohorts, severity of AKI with requirement of RRT and short-term renal recovery is important especially in critically ill cases of AAV (11, 12). Therefore, we here aimed to evaluate histopathological findings and predictive value of Berden's histopathological subgrouping and ARRS for the requirement of RRT during short-term clinical course in critically ill patients requiring intensive care treatment. Requirement of RRT during the short-term course of disease was associated with active glomerular lesions reflected by lower number of normal glomeruli, increased glomerular necrosis, and crescents. Berden's histopathological subgrouping into sclerotic and crescentic classes along with ARRS high-risk classification identified patients at risk for RRT requirement during the short-term disease course, both considered to also show the poorest long-term renal survival rates (4, 5). In contrast, presence of global glomerular sclerosis was superior to histopathological subgrouping and ARRS and the strongest negative predictor for recovery from RRT during the short-term course of disease in critically ill patients with AAV requiring intensive care treatment. Glomerular sclerosis is only included in Berden's histopathological subgrouping in cases with affected glomeruli above 50%, predicting long-term renal survival rates poorest in the sclerotic class. In their study, Berden et al. classified 13/100 (13%) patients in the sclerotic class (4). In contrast, only 3/46 (6.5%) patients could be classified as sclerotic class in our cohort. Possibly, a more acute and aggressive onset of severe AAV requiring RRT early at disease onset in our cohort resulted in fewer sclerotic glomeruli, as also reported in other cohorts (13). However, our observation that presence of sclerotic glomeruli associates with failure to recover from RRT in critically ill patients may implicate underestimation of glomerular sclerosis when histopathological subgrouping is used in this subgroup of severe AAV. This is in line with previous reports of renal recovery in patients requiring RRT over a 3-month period of time (14). Since severity of AKI, requirement of RRT, and short-term renal recovery in critically ill patients are associated with disease severity and clinical course of disease, this emphasizes the novelty and great relevance of our findings (6).

The main limitations of our study are being retrospective, different regimens of remission induction, the small patient number, and limited data on long-term renal survival rates. Nevertheless, our observations support that global glomerular sclerosis associates with failure to recover from RRT after initiation of remission induction therapy in cases of severe AAV requiring intensive care treatment. This new aspect requires further investigation in a prospective controlled setting for therapeutic decision making especially in this subgroup.

## Data Availability Statement

The original contributions presented in the study are included in the article/Supplementary Material, further inquiries can be directed to the corresponding author/s.

## Ethics Statement

The studies involving human participants were reviewed and approved by the Ethics committee of the University Medical Center Göttingen, Germany (no. 4/8/19). The patients/participants provided their written informed consent to participate in this study.

## Author Contributions

SH and BT conceived the study, collected and analyzed data, and co-wrote the first draft. DT collected and analyzed the data. SH and PS evaluated the histopathological findings. PK and MZ participated in the construction and editing of the manuscript. All authors contributed to the article and approved the submitted version.

## Conflict of Interest

The authors declare that the research was conducted in the absence of any commercial or financial relationships that could be construed as a potential conflict of interest.
