# Supplementary material for: Histopathological Findings Predict Renal Recovery in Severe ANCA-Associated Vasculitis Requiring Intensive Care Treatment
Source: Front Med (Lausanne). 2021 Feb 9;7:622028. doi: 10.3389/fmed.2020.622028 (PMC7900153; doi:10.3389/fmed.2020.622028)
Supplement: Supplementary file 1 [file Data_Sheet_1.PDF]

Histopathological findings predict renal recovery in severe ANCA-associated vasculitis requiring intensive care treatment

Supplementary Material

Samy Hakroush[1], Desiree Tampe[2], Peter Korsten[2], Philipp Ströbel[1], Michael Zeisberg[2,3], Björn Tampe[2]

[1]*Institute of Pathology, University Medical Center Göttingen, Göttingen, Germany*

[2]*Department of Nephrology and Rheumatology, University Medical Center Göttingen, Göttingen, Germany*

[3]*German Center for Cardiovascular Research (DZHK), Göttingen, Germany*

Corresponding author:

Björn Tampe, MD

Department of Nephrology and Rheumatology

University Medical Center Göttingen

Georg August University

Göttingen, Germany

Email: bjoern.tampe@med.uni-goettingen.de

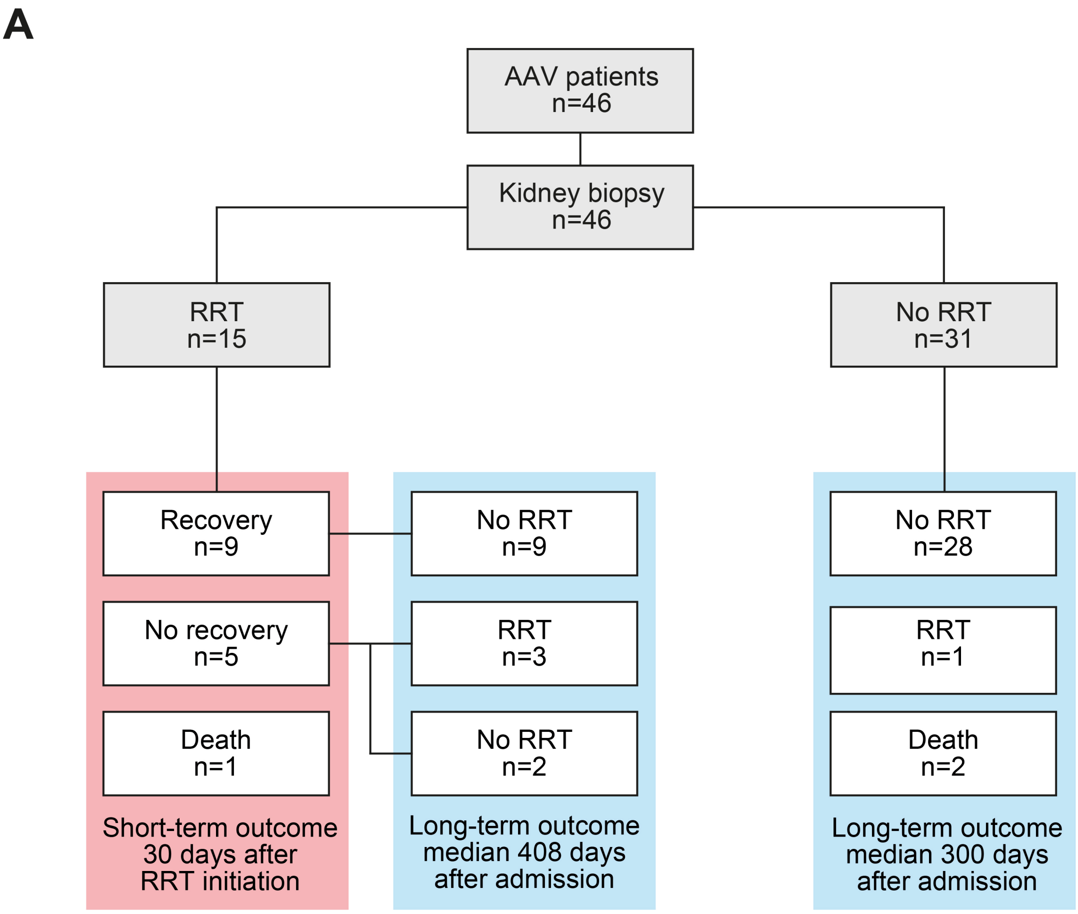

Supplemental Figure 1. (**A**) STROBE flow chart of patient disposition, RRT was performed intermittently in all cases. STROBE, Strengthening the Reporting of Observational Studies in Epidemiology.

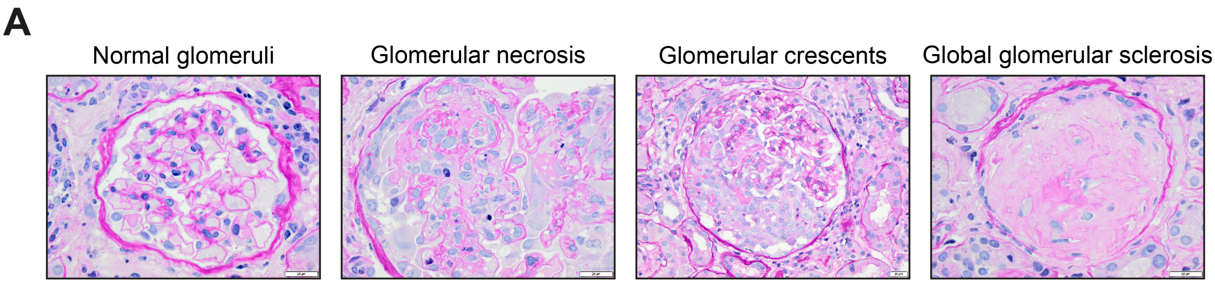

Supplemental Figure 2. (**A**) Representative images of glomerular findings in kidney biopsy specimens.

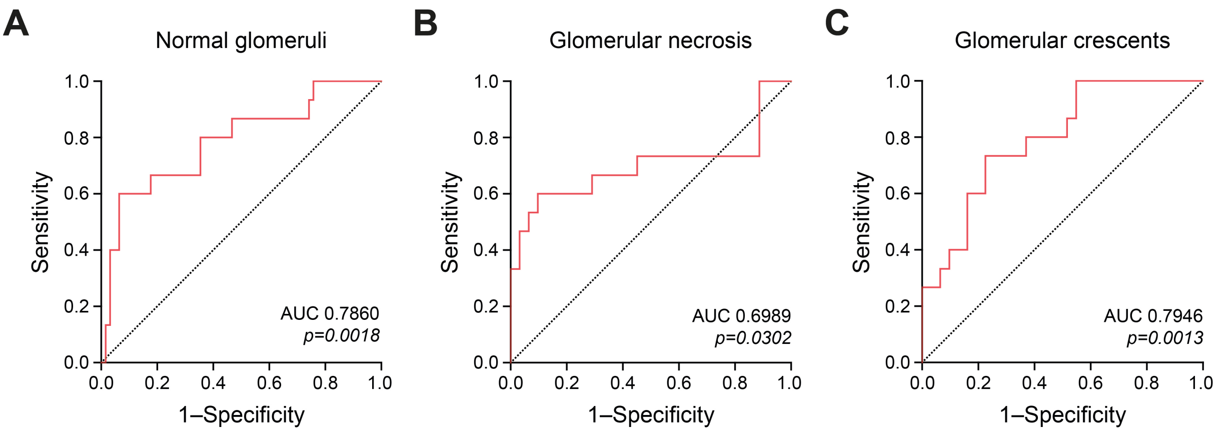

Supplemental Figure 3. (**A-C**) ROC analysis for each parameter for association with requirement of RRT within 30 days after admission are shown. AUC, area under the curve.

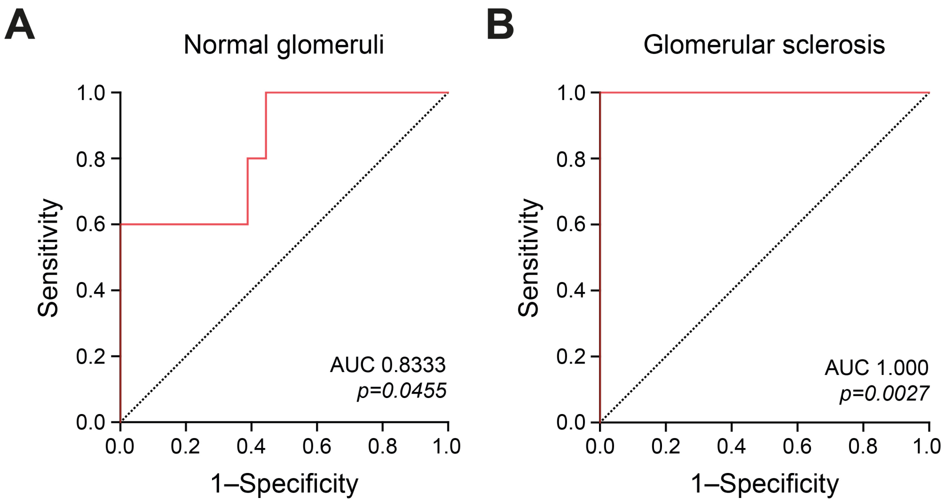

Supplemental Figure 4. (**A,B**) ROC analysis for each parameter for association with recovery from RRT within 30 days after RRT initiation are shown. AUC, area under the curve.
